# Natural Ingredients to Improve Immunity

**DOI:** 10.3390/ph16040528

**Published:** 2023-04-01

**Authors:** Amin Gasmi, Mariia Shanaida, Oleksandra Oleshchuk, Yuliya Semenova, Pavan Kumar Mujawdiya, Yana Ivankiv, Olena Pokryshko, Sadaf Noor, Salva Piscopo, Stepan Adamiv, Geir Bjørklund

**Affiliations:** 1 Société Francophone de Nutrithérapie et de Nutrigénétique Appliquée, 69100 Villeurbanne, France; 2 I. Horbachevsky Ternopil National Medical University, 46001 Ternopil, Ukraine; 3 School of Medicine, Nazarbayev University, 5/1 Kerey and Zhanibek Khandar Str., Astana 010000, Kazakhstan; 4 Inochi Care Private Limited, New Delhi 110017, India; 5 Institute of Molecular Biology and Biotechnology, Bahauddin Zakariya University Multan, Multan 60000, Pakistan; 6 Department of Nutritional Research and Development, Nutri-Logics SA, 9990 Luxembourg, Luxembourg; 7 Department of General Dentistry, Odessa National Medical University, 65000 Odessa, Ukraine; 8 Council for Nutritional and Environmental Medicine, 8610 Mo i Rana, Norway

**Keywords:** natural bioactive compounds, medicinal plants, vitamins, minerals, melatonin, propolis, probiotics, prebiotics, dosages, immunomodulators, antioxidants

## Abstract

The immune system protects the body from infectious agents such as bacteria, viruses, or fungi. Once encountered with pathogens or antigens, the innate and adaptive arms of the immune system trigger a strong immune response to eliminate them from the system and protect the body. Thus, well-balanced immunity is pivotal for maintaining human health, as an insufficient level of immune defense leads to infections and tumors. In contrast, the excessive functioning of the immune system causes the development of autoimmune diseases and allergies. Strong immunity requires adequate nutrition, dietary interventions, and sufficient intake of certain vitamins (vitamin C, vitamin D, and folic acid) and minerals (magnesium, zinc, and selenium). Therefore, nutritional and micronutrient deficiencies lead to compromised immunity. Several natural ingredients have shown potent immunomodulatory properties. The immune-enhancing properties of many plants and fungi are due to containing bioactive phytoconstituents such as polyphenols, terpenoids, β-glucans, vitamins, etc. Probiotics and prebiotics can be used as innovative tools to reduce intestinal inflammation and downregulate hypersensitivity reactions. Plant sources of melatonin, a multifunctional molecule with proven anti-inflammatory and immunomodulatory properties, have been discovered relatively recently. The bioactive compounds augment the immune response by directly increasing the cytotoxic activity of natural killer cells, macrophages, and neutrophils. Many phytoconstituents prevent cell damage due to their powerful antimicrobial, antioxidant, and anti-inflammatory properties. The present review attempts to understand the molecular mechanisms underlying the immune-enhancing properties of some bioactive compounds from plants, fungi, animals, microorganisms, and other natural sources.

## 1. Introduction

Humans are constantly exposed to various external influences, such as environmental pollutants or harmful pathogens, that affect homeostasis [1]. In recent decades, the rapid growth of the frequency of various infectious diseases, inflammatory conditions, allergies, cancers, and autoimmune disorders has brought much suffering to human beings. At the beginning of the 21st century, Parkin and Cohen [2] summarized that an insufficient level of immune defense leads primarily to the occurrence of severe infections or/and tumors, while the excessive functioning of the immune system causes the development of allergic or/and autoimmune diseases. Thus, well-balanced immunity is pivotal for maintaining human health [3]. Therefore, the importance of using natural substances with immunomodulating properties to prevent or augment the treatment of the abovementioned conditions is becoming quite obvious [4].

The immune system comprises a wide range of immune cells, various cytokines, and signaling pathways that protect the skin, intestinal tract, and respiratory tract from foreign invaders such as bacteria, viruses, and fungi. The host immune system has two arms: the innate immune system and the adaptive immune system [2]. While innate immunity lacks immunological memory, it is a hallmark of adaptive immunity [5].

Medicinal plants, beekeeping products, probiotics and prebiotics, melatonin, and other natural compounds with immunomodulating properties could explore new therapeutic ways to enhance immunity against various diseases [6,7,8,9]. Many vitamins and minerals are also very effective in supporting the proper functioning of immunity [7]. The high cost of synthetic drugs, their anticipated toxicity, and various adverse effects are undesirable for patients. In contrast, herbal substances as health promoters have gained increasing attention in both scientific circles and among consumers [10]. Some medicinal plants can exhibit immunomodulatory effects such as phagocytosis promotion and macrophage activation, modulation of cytokine secretion, immunoglobulin production, and lymphocyte proliferation [10]. In addition to the significant growth of interest in so-called superfoods (berries, nuts, green tea, sea products, and honey, among many others) in the era of the coronavirus pandemic [11], COVID-19 has prompted a search for new effective natural remedies for immunomodulation to prevent this disease. Natural phytotherapeutics were regarded as effective immune boosters in the case of COVID-19 disease [12]. Despite the progressive successes in developing various vaccines against COVID-19, the importance of natural substances as immune-enhancing agents cannot be underestimated [7]. Recently, herbal medicines have been considered the drugs of choice to enhance immunity in pre- and post-COVID-19 patients [13].

Plant-based natural ingredients promote health and help fight infections by boosting host immunity. Some phytoconstituents increase the proportion of beneficial gut bacteria, which are crucial for maintaining immunity. Recently, Rondanelli et al. described the pivotal role of vitamins (C, D), zinc, and *Echinacea* preparations in self-care for the prevention or treatment of common colds [14]. The natural ingredients of garlic (*Allium sativum*) boost immunity by increasing the functions of innate immune system cells such as lymphocytes, γδ-T cells, and natural killer (NK) cells, which kill invading pathogens [15]. β-Glucans are a category of naturally derived ingredients (from some plants, fungi, and bacteria) that modulate innate and adaptive immune responses. However, the immune response generated by β-glucans varies based on the structure [16]. The appropriate intake of some polyphenols, vitamins, and minerals helps boost immune functions and fight infections, including COVID-19 [17]. The immune-boosting properties of flavonoids help fight cancer, inflammation, and viral infections [18,19].

It should be noted that a balanced immune response also requires the suppression of unnecessary and harmful immune reactions. As it is known, dendritic cells (DCs) directly link the innate and adaptive immune systems, and their abnormal activation leads to chronic inflammation, rejection of transplants, and autoimmunity. It was revealed that quercetin, as a representative of flavonoids, can act as an immunosuppressant by inhibiting the activation of DCs [20]. Nowadays, scientists have concluded that using nanoformulations helps successfully solve the problem of poor aqueous solubility and low bioavailability of some natural constituents such as curcumin, quercetin, gingerol, etc. [21,22,23]. Nanodelivery of drugs makes it possible to provide targeted delivery of bioactive components and helps significantly reduce their dosage.

Micronutrients, such as vitamin C, also improve immune functions by augmenting chemotaxis and phagocytosis and generating free radicals by immune cells [24]. Similarly, vitamin D increases the Th2 cell response and suppresses Th1 cell activation [25]. Overall, deficiencies in certain vitamins and minerals can lead to the weakening of the immune system [26]. It should be also mentioned that propolis, a famous beekeeping product, is widely used for managing upper respiratory tract infections, which occur when the level of immunity decreases [8,27]. It is also worth adding that research in recent years has revealed that appropriate administration of the hormone melatonin plays a significant role in improving immunity [28,29].

This review attempts to describe the modern approaches to the immune-boosting roles of a variety of natural ingredients from different sources and their use in managing infections or other diseases related to the dysfunction of the immune system.

## 2. Medicinal Plants as Immunomodulators

Several botanical families comprise many plants with proven immunomodulating properties, including *Asteraceae*, *Rutaceae*, *Araliaceae*, *Zingiberaceae*, *Alliaceae*, *Lamiaceae*, etc. [30,31,32,33]. For example, the immune-stimulating properties of *Echinacea*, ginger, and fresh citrus juice have been well-known worldwide for several decades [1,32,34,35]. In recent years, an increasing number of herbs with immunostimulating properties have been researched and discovered for official medicine based on the study of plant species from different parts of the globe. Herbal preparations are regarded as effective and inexpensive natural immunomodulators [1,36,37,38]. Williamson summarized that the molecular pharmacology of herbal drugs is a tremendous challenge, as phytoextracts are multicomponent substances with multileveled modes of action compared to synthetic pharmaceuticals [39].

Thomford et al. [40] noted that modern approaches to medicine in the 21st century, as a rule, require the isolation and purification of one or two active compounds from medicinal plants. However, the isolation of an active compound could lead to the loss of its pharmacological activity [40]. For instance, the effectiveness of multi-component herbal prescriptions in traditional Chinese medicine is highly appreciated by modern health care practitioners. It should be noted that individually, many of the ingredients in these combinations do not possess therapeutic activities because of the simultaneous and synergistic action of several bioactive compounds [40]. The modern technologies such as metabolomics, genomics, proteomics, transcriptomics, and computational strategies open the way for choosing better drug candidates with immunomodulatory effects extracted from medicinal plants [41].

According to Boozari and Hosseinzadeh, the consumption of herbal substances prepared from *Echinacea* spp., *Zingiber officinale*, *Camellia sinensis*, *Hypericum perforatum*, *Allium sativum*, *Glycyrrhiza glabra*, and *Nigella sativa* can significantly improve the immune response in the fight against the novel coronavirus COVID-19 [42]. It was found that phytoconstituents from such ayurvedic medicinal plants as Ashwagandha (*Withania somnifera*), Tulsi (*Ocimum sanctum*), and Giloy (*Tinospora cordifolia*) were effective COVID-19 virus proteases [30,43]. Coriander (*Coriandrum sativum*) was regarded as the most commonly used ingredient in homemade remedies in Sri Lanka during the COVID-19 pandemic [44]. Herbal preparations are regarded as effective and inexpensive natural immunomodulators [1]. Recent findings of Al Kury et al. [45] demonstrate that an extract from the aerial part of *Xanthium spinosum* (*Asteraceae* family) has significant anticancer and immunomodulatory effects.

Many polyphenols, terpenoids, sulfur-containing compounds, polysaccharides, and some alkaloids possess the ability to enhance immunity [33,46,47]. Venter et al. [48] concluded that unsaturated omega-3 fatty acids from plants sources also play an important role in the maintenance of immunity in the context of autoimmune and allergic diseases.

However, the lack of reliable standardization of bioactive ingredients and rigorous tests for efficacy constitute a significant obstacle regarding the poor progress in research studying the immunomodulatory effects of herbal remedies [1,36]. Recently, Sun et al. [49] concluded that increased solubility, bioavailability, and efficacy of herbal formulations with immunomodulatory effects can be achieved using nano-engineered delivery systems. Chaudhary et al. [21] found that the synthesis of new antimicrobial drugs using ‘green nanoparticles’ from plant extracts has attracted a lot of attention from scientists in recent years.

### 2.1. Coneflower (Echinacea ssp.)

Coneflower (*Echinacea)* is a genus of herbaceous plants (*Asteraceae* family) originating from North America. Three species (*E. purpurea*, *E. pallida*, and *E. angustifolia*) have been used by Native Americans for the treatment of respiratory infections and inflammatory conditions, including bronchitis and inflammation of the mouth [1]. Phytopreparations from the coneflower rhizome with roots or herbs are also widely used in the 21st century in the pharmaceutical industry. They are the best-selling herbal drugs in the USA and Europe as effective natural immunomodulators [50]. 

The quality of *E. purpureae herba* and *radix* may be determined by the content of cichoric acid, which is regarded as their main bioactive compound, while dominating echinacoside is characteristic of the *E. angustifoliae* and *E. pallidae radices* [1]. In addition to these phytoconstituents, several other bioactive chemicals have been identified in *Echinacea* species. Thus, their N-alkylamides also possess significant immunomopharmacological effects [51]. Primary metabolites such as polysaccharide arabinogalactan from *E. purpurea* could also enhance macrophage activation [52]. It was suggested that treatment with standardized *Echinacea* extracts at 2.4 g/day over 120 days is beneficial for prophylactic use and a dose of 4.0 g/day during acute stages of colds [14]. The extract from the fresh aerial part of *E. purpurea* (10–250 µg/mL) could effectively modulate the human Jurkat T-cell cytokine response through interleukin-2 and interferon-gamma cytokine secretions [53]. 

In vitro studies of the ethanolic extract prepared from *E. purpurea* showed its significant effect on inhibiting highly pathogenic SARS-CoV-2 [54]. Vimalanathan et al. [55] showed the broad antiviral actions of *E. purpurea* against SARS-CoV-2. Recently, Kolev et al. [56] demonstrated in randomized clinical studies the ability of *E. purpurea* hydroethanolic extract to prevent respiratory tract viral infections during the COVID-19 pandemic in the long term. 

### 2.2. Artemisia afra/annua

*Artemisia* (*Asteraceae* family) is a widely known genus of immense medicinal value. It comprises almost 500 species of plants. The famous antimalarial drug artemisinin was isolated from the Chinese species sweet wormwood (*A. annua*). The significant interest in this compound has increased in the past three years, due to the COVID-19 pandemic, as it also possesses antiviral effect [57].

Several volatile secondary phytoconstituents from *Artemisia* species are part of traditional medicinal systems worldwide. In the South African region, *A. afra* is one of the most common and widely used herbs to manage diabetes, malaria, and cough [58]. *A. afra* also possesses powerful antimicrobial properties due to the presence of bioactive terpenoids. The essential oils of *A. afra* showed antimicrobial properties against 41 bacterial strains, indicating broad antimicrobial activity, which favors its use as a preservative in the food and cosmetic industries [59]. Three compounds of *A. afra*, scopoletin, betulinic acid, and acacetin, have shown potent antimicrobial activity against a wide range of gram-negative and gram-positive bacterial species and *Candida albicans* [60]. Appalasamy et al. reported the antimicrobial properties of *A. annua*. However, the compounds in *A. annua* were not found to be effective against *Candida albicans* [61]. The extract of *A. annua* also showed dose-dependent inhibition of tumor necrosis factor-alfa (TNF-α) in activated neutrophils. At 25, 10, and 5 µg/mL concentrations, *A. annua* inhibited TNF-α secretion by 89%, 54%, and 38%, respectively. It also inhibited the secretion of prostaglandin E2 by activated neutrophils [62]. It is pertinent to mention that some studies have reported hepatotoxic effects of *A. annua,* where consumption of *A. annua* herbal tea was associated with acute cholestatic hepatitis. Therefore, the authors recommend its discontinuation for use in malaria due to its ineffectiveness and toxic effects on the liver [63]. A 2012 report by the European Food Safety Authority classified several *Artemisia* species in a category that “*contain naturally occurring substances of possible concern for human health when used in food and food supplements*.” Moreover, the Medicine Control Council of South Africa has also banned the use of *A. afra* for the treatment of malaria due to its lack of efficacy [64].

### 2.3. Ginseng (Panax ginseng)

Numerous studies have demonstrated that ginseng (*Panax ginseng*) as well as several other species from the *Araliaceae* family can modulate the immune system, thereby preventing many health disorders and treating immunity-related diseases [48]. Many isolated ginsenosides (including Re, Rf, and Rg2) possess noticeable positive effects on innate and adaptive immunity [65].

Interestingly, the immune-stimulating properties of ginseng are modulated by the fermentation process. For instance, the fermentation of an ethanol extract of red ginseng by *Lactobacillus plantarum* M-2 enhanced the immunological benefits of red ginseng. In the mouse model, intraperitoneal injection of 500 µg fermented red ginseng (FRG-E) was more effective in reducing lung metastasis than nonfermented red ginseng (NFRG-E) (81.1% v/s 66.9%). Moreover, FRG-E improved immunity in healthy subjects by increasing the levels of IgA and IgG. Surprisingly, NRFG-E reduced IgA and IgG levels in subjects compared with the baseline values (5.14 mg/mL v/s −14.5 mg/mL at one week), indicating improved immunological activity of FRG-E compared with NFRG-E [66]. 

A study conducted in patients with non-small cell lung cancer demonstrated that ginseng polysaccharides (GPS) alter the immune response of T helper cells (Th1 and Th2) compared to DCs alone. Both the GPS + DC and DC groups showed higher levels of Th1 cytokines (INF-γ and IL-2) and reduced levels of Th2 cytokines (IL-4 and IL-5), leading to a higher ratio of Th1/Th2 cytokines. Importantly, patients in the GPS + DC group showed higher immune functions than those in the DC alone group, indicating the potent immunomodulatory effects of GPS [67]. 

A randomized, placebo-controlled, parallel, double-blind study on 72 healthy subjects demonstrated that ginseng polysaccharide Y-75 (6 g/day) significantly increased immune functions compared to the control group. Intake of Y-75 enhanced NK cell activity by 35.2% and 40.2% from baseline values after 8 and 14 weeks of treatment, respectively. Similarly, the phagocytic activity of peripheral blood cells increased significantly by 25.2% and 39.4%. Moreover, serum TNF-α levels showed 38.2% and 44.5% increases at 8- and 14-weeks posttreatment, respectively. Importantly, Y-75 was found to be safe and efficacious in improving immune functions [68]. Sun et al. demonstrated that oral administration of 200 and 400 mg/day ginseng polysaccharides in cyclophosphamide-induced immunocompromised mice significantly improved immunity by increasing NK cell cytotoxic activity and increasing the secretion of perforin and granzyme [69].

### 2.4. Spices

A lot of different spices are widely used in cooking. As it turned out, some of them have a significant effect on the functioning of the immune system. Many spices that are commonly known as medicinal plants possess immune-enhancing properties. Thus, the rhizome of ginger (*Zingiber officinale*) contains dozens of compounds with antioxidant activities that could be used to treat various inflammations as well as modulate metabolic biomarkers. Karimi et al. [70] demonstrated that four capsules of ginger extract (750 mg) were used daily for six weeks by obese women with breast neoplasms to reduce systemic inflammation and metabolic syndrome. Yücel et al. [23] described gingerols’ anti-inflammatory and immunomodulatory potential and their nanoformulations. The presence of a significant amount of eugenol in the essential oil as well as polyphenols (including apigenin, quercetin, and ferulic acid) in the aerial parts of ‘Holy basil’ or ‘Tulsi’ (*Ocimum sanctum*) from the *Lamiaceae* family could be the substantial reason for its excellent anti-inflammatory and immunostimulating activity [71]. The main biocompounds of garlic (*Allium sativum*) belong to *γ*-glutamyl cysteine derivatives, allyl methyl cysteine, and diallyl thiosulfinate compound families [72]. Allicin orally applied at doses of 3–9 mg/kg/day reduced parasitemia in mice infected with *Plasmodium malaria* and prolonged their survival by improving host immune responses [73]. The cumin (*Nigella* ssp.) seeds contain thymoquinone, β-elemen, dihydrofarnesol, and other bioactive terpenoids [74] that exhibit significant antioxidant and anti-inflammatory effects. Clinical trials revealed that *Nigella* seed oil has substantial potential to alleviate inflammation in the case of rheumatoid arthritis [74]. 

**Figure 1:**
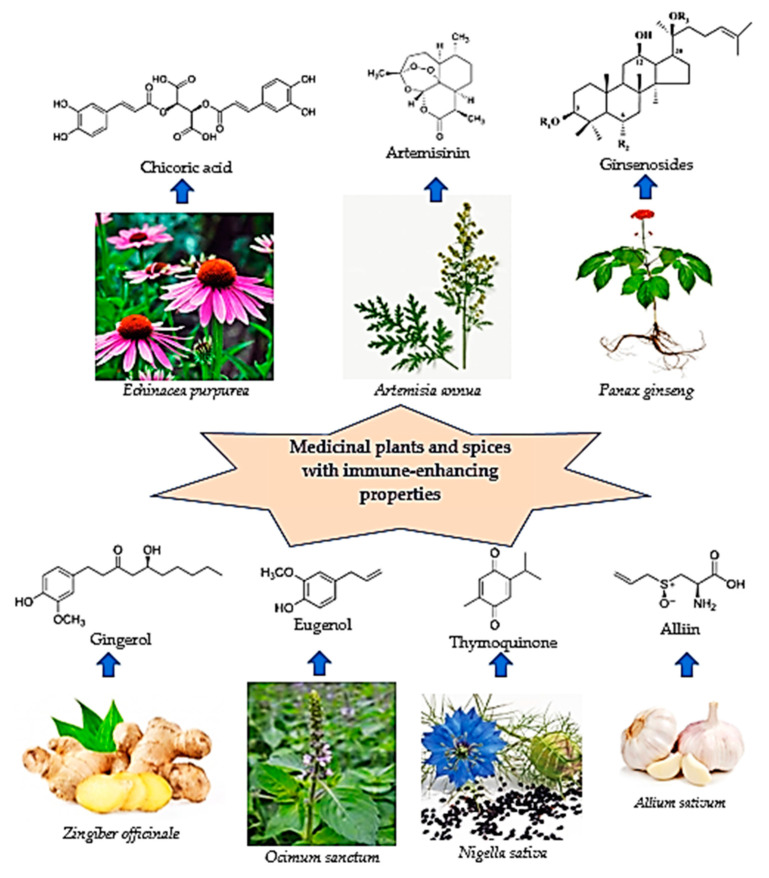
The most important medicinal plants and spices with immunomodulatory activity.

Figure 1 presents the most important medicinal plants and spices with immunomodulatory effects.

Thus, a lot of herbs are quite effective and inexpensive natural sources of phytoconstituents with immune-enhancing properties. Some polyphenols, terpenoids, sulfur-containing compounds, polysaccharides, etc., from medicinal plants and spices possess significant potential to improve immunity.

## 3. Selected Natural Compounds with Immunomodulating Properties

Quercetin, curcumin, luteolin, and other polyphenols demonstrate prominent immunostimulatory actions on macrophages as well as the ability to increase the proliferation of T cells and B cells [75]. Polyphenols can also reduce inflammation by suppressing proinflammatory cytokines and causing immunomodulatory effects against autoimmune diseases and allergic reactions [76]. According to Mollazadeh et al. [77], the positive effects of curcumin on human immunity could be related to its ability to increase IL-10-mediated effects. 

Among other well-known components with an immunomodulatory effect, it should be mentioned propolis as a beekeeping product, melatonin as a representative of the class of neurohormones, or some polysaccharides of plants and mushrooms. Xylitol, as a non-digestible carbohydrate of natural origin, beyond its significant role in maintaining dental health, also modulates the immune response of the body [78,79]. It contributes to a reduced respiratory tract infection risk [79].

Below are the results of scientific research on the immunomodulatory potential of some biologically active components that have different resource origins. 

### 3.1. Quercetin

Quercetin is a flavonoid with the chemical name 3,3′,4′,5,7-pentahydroxyflavone. Quercetin is not synthesized in the human body, and it is found in several plant species. Its anti-inflammatory, antioxidant, antiviral, and antibacterial properties are well reported in the literature [75,80].

In a randomized, double-blind, placebo-controlled clinical trial conducted in 50 women with rheumatoid arthritis, the intervention of quercetin (500 mg/day) for eight weeks significantly improved disease symptoms by reducing pain, disease activity, and plasma levels of hs-TNFα compared with the placebo group. Although quercetin supplementation affected the erythrocyte sedimentation rate, the difference was insignificant [81]. In a meta-analysis, quercetin supplementation significantly reduced CRP levels, total cholesterol, and low-density lipoprotein in patients suffering from metabolic disorders. However, no effect on IL-6, TNF-α, or high-density lipoprotein was observed [82]. However, a randomized, crossover study in 20 athletes showed that intake of four quercetin-based chews 15 min before a 2 h run did not reduce post-exercise inflammation or immune changes caused by increased levels of various cytokines, such as GM-CSF, IL-2, IL-6, IL-8, CRP, TNF-α, and IL-1β. The four quercetin-based chews provided to runners were made using 1000 mg quercetin, 1000 mg vitamin C, 400 mg each of isoquercetin, eicosapentaenoic acid, and docosahexaenoic acid, 120 mg epigallocatechin 3-gallate, and 40 mg niacinamide. Although quercetin levels significantly increased in the quercetin-based chews group, no statistically significant difference was observed in the postexercise inflammation levels compared to the placebo group [83]. 

**Figure 2:**
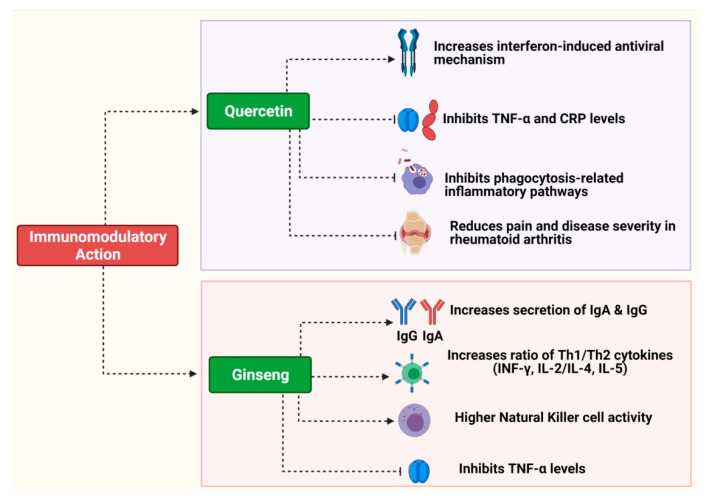
Immunomodulatory activities of quercetin and ginseng.

Heinz et al. conducted a double-blinded, placebo-controlled, randomized trial to evaluate the impact of long-term supplementation of quercetin on the innate immune system. Supplementation of quercetin (500 mg/day and 1000 mg/day) to adult female subjects for 12 weeks had no significant impact on plasma IL-6 and TNF-α levels, and no significant impact on NK-cell activity and granulocyte phagocytosis was observed compared to the placebo group, indicating no direct impact on innate immunity and inflammation [84]. Figure 2 presents an overview of the immunomodulatory properties of quercetin and preparations from ginseng.

### 3.2. Propolis

Propolis is a highly viscous and resinous natural product produced by honeybees and possesses substantial pharmacological and biological properties [8]. Propolis is recommended for managing upper respiratory tract infections, flu, colds, wound healing, fighting bacterial, viral, and fungal infections, and treating acne or burns [27]. The antioxidant properties of propolis primarily contribute to its medicinal properties. A meta-analysis and systemic review of six studies with 406 participants concluded that propolis use significantly lowered serum TNF-α and CRP levels in study participants [85]. A double-blind, placebo-controlled clinical trial in 50 type 2 diabetes mellitus patients showed that the treated group receiving Iranian propolis (1000 mg/day) for 90 days showed a significant reduction in serum levels of CRP, TNF-α, glycosylated hemoglobin, and insulin [86]. A randomized, double-blind trial conducted in 60 elderly subjects (average age: 72.8 years) living at higher latitudes (2260 m above sea level) demonstrated that propolis intake (0.8 g; 24 months) significantly reduced serum IL-1β and IL-6 levels, but serum TNF-α levels were not statistically significant. However, the propolis-treated group showed significantly higher levels of TGF-β1 [87].

Similarly, in an 18-week randomized controlled study involving 33 type 2 diabetes mellitus patients, supplementation with 900 mg/day of Brazilian green propolis significantly reduced serum TNF-α levels but significantly increased serum IL-1β and IL-6 levels. Moreover, serum antioxidant capacity and total polyphenol concentrations were significantly higher in the propolis-treated group, while serum carbonyls and lactate dehydrogenase activity were significantly lower [88]. Diabetic foot is a major diabetes-associated complication. The use of propolis has been proposed to heal diabetic foot ulcers due to its anti-inflammatory, antioxidant, and wound-healing properties. A randomized trial conducted on 31 diabetic foot ulcer patients showed that propolis spray (3%) applied to wounds for eight weeks reduced the wound area by 4 cm^2^ and facilitated wound healing via increased tissue deposition at the wound site.

Moreover, propolis reduced TNF-α but increased IL-10 and the glutathione/glutathione disulfide ratio. Therefore, propolis may be used as an adjuvant therapy in managing diabetic foot due to its anti-inflammatory and antioxidant potential [89]. A meta-analysis of six studies comprising 406 participants demonstrated that propolis intake significantly reduced serum levels of CRP and TNF-α [85]. In a double-blind, randomized, placebo-controlled clinical trial conducted on 31 dengue hemorrhagic fever patients in Indonesia, patients were given two capsules of 200 mg of Propoelix^TM^ three times a day for seven days. The PropoelixTM-treated group showed faster recovery, as evidenced by increased platelets compared to the placebo group. Moreover, the treated group had a significantly lower TNF-α level on day seven than the placebo group. Overall, the treated group had lower hospitalization stays and better recovery [90].

### 3.3. Glucans

β-Glucan is a dietary fiber and a long-chain polymer of D-glucose, where D-glucose moieties are joined through glycosidic linkages. β-Glucans are a common constituent of the cell walls of several bacteria, algae, and fungi (*Saccharomyces cerevisiae, Aspergillus*, *Ganoderma applanatum,* and *Inonotus obliquus*), as well as cereals and grains (barley, wheat, oats, and rye) [29,60]. It was found that β-glucan contains various types of glycosidic linkages, β-(1,3)/β-(1,4)/β-(1,6), which vary depending on the source [91]. β-Glucans are a dietary source of soluble fermentable prebiotic fibers that provide a substrate for host microbiota within the large intestine, producing short-chain fatty acids and increasing fecal bulk.

It has been reported that β-glucan improves host immunity by activating the complement system and increasing the cytotoxic activity of macrophages and NK cells. Moreover, the interactions of β-glucan with several cell surface receptors, such as complement receptor 3, dectin-1, and lactosylceramide selected scavenger receptors, also trigger immune functions [92].

In animal models, beta 1,3/1,6 glucan from the mushroom reishi (*Ganoderma lucidum*) improved immunity by inducing the expression of IgA or IgG in serum and small intestinal fluid. Moreover, it also increased the expression of the poly-Ig receptor in the small intestine and promoted NK cell activity [93]. Water-insoluble β-(1→3)-D-glucan extracted from *G. lucidum* displayed potent anti-inflammatory properties in lipopolysaccharide (LPS)-induced Raw 264.7 cells by downregulating iNOS and TNF-α mRNA expression and inhibiting IκBα and JNK1/2 phosphorylation. At the molecular level, the anti-inflammatory activity of β-(1→3)-D-glucan was due to the inhibition of the NF-κB and JNK MAPK pathways, with TLR2 receptors playing a central role [94]. 

It has been demonstrated that the chemical structure of glucans (α or β) alters the bioactivity of glucans. For instance, β-(1→6), β-(1→3), (1→6), and α-(1→3) glucans were purified from a polysaccharide-enriched extract from *Lentinula edodes* (shiitake mushrooms). However, only the β-(1→6) and β-(1→3), (1→6) fractions displayed antioxidant properties against DPPH free radicals and lowered IL-1β and IL-6 secretion in LPS-stimulated THP-1/M cells, indicating the crucial role played by chemical structure in the biological activity of glucans [95]. In a similar observation, Murphy et al. reported that the extraction process of β-glucans from the same source also creates a difference in immunomodulatory properties. In this study, the “in-house” extract of the edible mushroom (IHL) *Lentinula edodes* was compared with commercially available (Carbosynth-Lentinan, CL) lentinan extract. The CL extract is rich in α-glucans and contains lower concentrations of β-glucans. In human alveolar epithelial A549 cells, both IHL and CL extracts reduced NF-κB activation at lower doses, but the IHL extract was more effective in lowering NF-κB activation. However, the CL extract was more potent in inhibiting the release of proinflammatory cytokines, such as IL-2, TNF-α, IL-22, IL-8, and IL-6, than the IHL extract. These findings demonstrate that β-glucans from the same source can have different immunomodulatory and pulmonary protective properties, which may have implications for treating disorders caused by a cytokine storm in COVID-19 [96]. 

Gaullier et al. conducted a double-blind, crossover, placebo-controlled study in healthy Caucasian adults to understand the bioactivity of (1–6,1–3)-beta-glucan (lentinan) from *Lentinula edodes*. In this study, adults were either given lentinex (2.5 mg/day) or a placebo for six weeks. After a four-week washout period, alternate supplementation was given for an additional six weeks. The results demonstrated that both groups showed a higher number of NK cells and circulating B cells. However, no difference in the levels of antibodies, cytokines, and complement proteins was observed. Lentinex supplementation was safe and well tolerated by the study participants [97]. Gordon et al. reported that a combination of lentinan and didanosine increased the number of CD4 cells and neutrophil activity in some HIV patients [98]. 

*Cordyceps* is a well-known Chinese fungus and possesses powerful immune-boosting properties. An extract from *Cordyceps* mycelium at a dose of 1.68 g/day for eight weeks significantly enhanced the cytotoxic activity of NK-cell activity (38.8  ±  17.6% increase) in healthy human subjects (n = 40) compared to the control group (n = 39) [99]. In another study, the aqueous and alkaline extracts of *Cordyceps militaris* mushrooms containing β-D-Glcp (1→3)-linked polysaccharides were studied for their immunomodulatory properties. The aqueous extracts stimulated the expression of various cytokines (TNF-α, IL-1β, COX-2) in THP-1 macrophages, while the alkaline extract did not elicit any immune response. However, alkaline did show potent anti-inflammatory activity in LPS-stimulated cells by inhibiting proinflammatory genes. Furthermore, the purified β-(1→3)-D-glucan from *C. militaris* was the most potent anti-inflammatory agent under in vivo conditions and reduced the migration of total leucocytes but did not affect neutrophil migration [100]. A clinical study conducted in healthy male adults (n = 39) demonstrated that capsules of *C. militaris* (dose: 1.5 g/day, 4 weeks) significantly enhanced cell-mediated immunity. The treated group displayed higher NK cell activity and lymphocyte proliferation index and induced the secretion of IFN-ϒ by Th1 cells compared to the placebo group. Therefore, *C. militaris* effectively enhanced cell-mediated immunity in adults without causing side effects [101].

**Figure 3:**
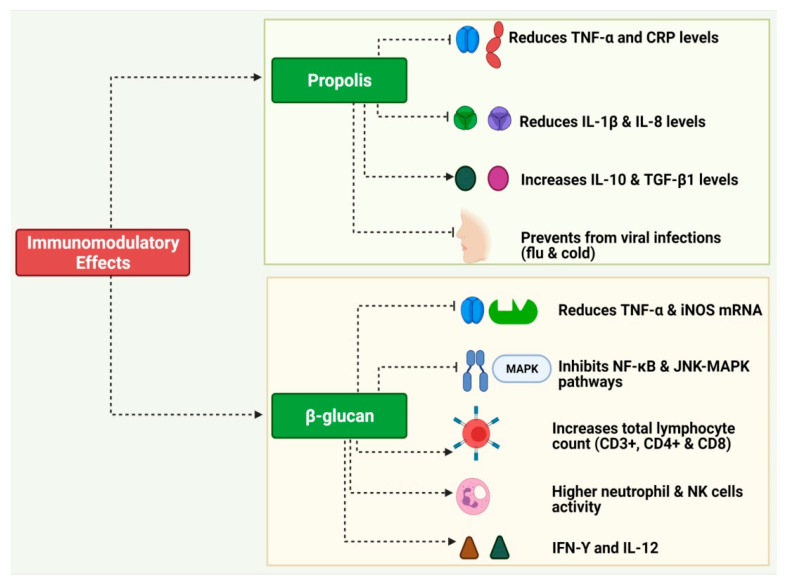
Immunomodulatory activities of propolis and β-glucans.

Lehne et al. reported that oral supplementation with 400 mg/day soluble branched yeast β-1,3-D-glucan derived from the *S. cerevisiae* cell wall for four days significantly increased the salivary concentrations of IgA (65·8 ± 29·4 v/s 105·4 ± 73·9 ug/mL). The 400 mg/day dose was well tolerated, and no adverse events were reported [102]. In a double-blind placebo-controlled trial, oyster mushrooms (*Pleurotus cornucopiae*) containing a β-glucan concentration of 24 mg/meal for eight weeks increased the serum levels of IFN-ϒ and IL-12, whereas IL-10 and IL-13 did not change considerably. Moreover, the treated group showed increased activity of NK cells. It may help fight infection and cancer due to its immune-boosting properties [103]. Figure 3 presents an overview of the immunomodulatory properties of propolis and β-glucans.

### 3.4. Melatonin

Melatonin is a multifunctional molecule known as an anti-inflammatory agent, immunoregulator, and antioxidant that might be beneficial and broadly applicable to treat a variety of proinflammatory diseases by inhibiting the immune response and reducing the production of proinflammatory cytokines [104]. It is a hormone that is an indole derivative of serotonin [28,105].

For a long time, melatonin was considered an animal neurohormone, and only at the end of the 20th century were discovered various plant sources of phytomelatonin [106]. The richest sources of melatonin are the seeds of *Coffea arabica* (6800 ng/g) and black pepper (1093 ng/g) [106]. It has been found also in cherries, grapes, pistachio, olives, walnuts, tomatoes, maize, rice, and some medicinal plants (*Curcuma longa*, *Tanacetum parthenium*, *Hypericum perforatum*, etc.).

Previous studies have shown that melatonin, a potent scavenger of reactive oxygen species and a potent inhibitor of myeloperoxidases, inhibits nuclear factor kappa B (NF-κB) signaling pathways, which play an important role in reducing the expression of inflammatory genes, including the signal transducer and activator of transcription expressed in T cells and GATA-binding protein 3, an apoptosis-associated speck-like protein containing the recruitment domain of caspase and caspase-1 [107]. Reviews highlight the important role of melatonin as a potential adjuvant in the treatment of COVID-19 due to its ability to influence mechanisms that alter immune regulation; melatonin has been included as a preventive and therapeutic option against COVID-19 along with vitamin D, vitamin C, zinc, and selenium [108]. These nutrients show synergistic effects and play a central role in maintaining the function and integrity of the immune system, so even a minor deficiency can impair metabolism and immune responses [105,109].

Melatonin has a regulatory effect on the immune system and directly enhances the immune response by increasing the proliferation and maturation of cells (T and B lymphocytes, granulocytes, and monocytes), enhancing antigen presentation in macrophages. The additional use of melatonin is associated with a significant decrease in the levels of the proinflammatory cytokines TNF-α, IL-1β, and IL-6 [110]. Considering the high effectiveness of oral melatonin as an adjuvant therapy added to standard treatment, the safety, and availability of the drug, it is suggested to conduct additional studies with larger sample sizes and longer observation periods on the use of melatonin for the prevention of complications and the treatment of COVID-19 patients of varying degrees of severity.

Therefore, a lot of in vitro, in vivo, and clinical studies convincingly demonstrate the significant immunomodulatory activities of quercetin and other polyphenols, as well as melatonin, β-glucans, and propolis, which have different resource origins and various mechanisms of immunomodulatory effects.

## 4. Micronutrients: Vitamins and Minerals

Many micronutrients play a crucial role in augmenting immune function and acting as immune boosters [111]. Some dietary micronutrients have been recognized as key components of the world-famous Mediterranean diet [112]. Each stage of an adequate immune response requires many specific elements, including zinc, selenium, magnesium, and vitamins, which play vital, often synergistic roles. As is known, many vitamins can be obtained from natural sources. For instance, a lot of fresh fruits and vegetables contain high amounts of vitamin C. However, some vitamins have a synthetic origin nowadays.

The daily demand for the consumption of micronutrients for proper functioning may be higher than the recommended dietary allowances [113]. As it was revealed recently, micronutrients are important immunomodulatory tools for COVID-19 management [75].

### 4.1. Vitamin C

Vitamin C, or ascorbic acid, is well known for its anti-inflammatory properties. It can increase the synthesis of cortisol and vasopressors and absorb free radicals affecting the functioning of leukocytes through the extracellular traps of neutrophils, thus strengthening the arsenal against various pathogens, including viruses and bacteria. However, there is still considerable controversy regarding vitamin C supplementation in various meta-analyses and multiple reviews due to the varied methodologies of the studies and the evaluation of the clinical efficacy of vitamin C use [114]. For example, vitamin C from fresh fruits and vegetables reduces cellular oxidative stress, improves immune functions by enhancing innate and adaptive immune responses, and acts as a cofactor for several enzymatic reactions. Moreover, the accumulation of vitamin C in neutrophils enhances chemotaxis and phagocytosis activity, leading to the killing of bacterial cells. It also promotes the clearance of spent neutrophils from the infection site and prevents tissue damage [24]. The preventive role of vitamin C in the common cold is well documented. Studies have shown that vitamin C supplements (1–2 g/day) reduce the severity of the common cold in children and adults.

Vitamin C concentrations are depleted during viral infections, and vitamin C deficiency is associated with postherpetic neuralgia. In 67 participants suffering from shingles, the intravenous administration of vitamin C (Pascorbin^®^ 7.5 g/50 mL) for two weeks in combination with standard therapy reduced herpes zoster-associated pain and other complaints, indicating the beneficial effects of vitamin C during viral infections [115]. In a randomized clinical trial conducted in 200 patients, supplementation with vitamin C and vitamin E in combination with triple therapy of antibiotics (lansoprazole + amoxicillin + clarithromycin) for 14 days increased the clearance rate of *Helicobacter pylori* by increasing antibiotic effectiveness and augmenting immune functions [116].

Network meta-analysis compared different doses of vitamin C (<6 g/day), high doses of vitamin C (<12, ≥6 g/day), very high doses of vitamin C (≥12 g/day), glucocorticoids (<400 mg/day of hydrocortisone), vitamin B1, and combinations of these drugs with placebo or usual treatment, and discovered that there were no significant differences in early mortality in adults with sepsis or septic shock between treatment and placebo/usual treatment or between treatments and found no evidence that vitamin C affects organ dysfunction or duration of stay in the intensive care unit [117].

### 4.2. Vitamin D

As it is known, vitamin D can be synthesized in the human skin in the sun and also enter the body thorugh the process of nutrition. The main natural sources of vitamin D are fish oil, cod fish liver, egg yolk, butter, and some fungi [118]. 1,25-Dihydroxyvitamin D, the active form of vitamin D, exerts anti-inflammatory effects through the presence of immune cells, including B and T cells, DCs, and macrophages expressing the vitamin D receptor and 1α-hydroxylase. It was found a significant association between vitamin D deficiency and increased incidence or exacerbation of infectious diseases and inflammatory autoimmune diseases. However, the impact of vitamin D on the treatment and prevention of infectious diseases and the relationship between inflammatory diseases and vitamin D are still controversial [119].

There is increasing evidence that vitamin D is physiologically important for the human host’s defense against bacterial and viral infections through vitamin D innate immune signaling mechanisms, including the production of cytokines, antimicrobial proteins, and pattern recognition receptors, and that vitamin D supplementation can combat viral infections, including those caused by SARS-CoV-2, which is supported by increasing clinical evidence of the beneficial effects of vitamin D supplementation in cases of vitamin D deficiency, which is common in most countries in North America and Europe and is particularly common in nursing homes, which are severely affected by the COVID-19 pandemic [120].

Based on recent studies, it is reasonable to avoid vitamin D deficiency in the population to maximize innate and adaptive immunity and prevent adverse cardiovascular outcomes during the COVID-19 pandemic by supplementing with vitamin D at doses recommended by the Endocrine Society to maintain 25-hydroxyvitamin D concentrations in blood serum at a level of at least 30 ng/mL, which can help reduce the risk of SARS-CoV-2 infection and its severe consequences, including mortality [121]. In a study involving 100 participants (50 with COVID-19 and 50 healthy subjects), it was noted that patients with COVID-19 had significantly lower serum 25-hydroxyvitamin D levels than healthy controls [23.10 ± 10.89 vs. 32.06 ± 17.22, *p* = 0.0024]. Additionally, the total numbers of lymphocytes, TCD4+, TCD8+, and NK cells were significantly decreased in patients with COVID-19 (*p* < 0.0001), and IL-12, IFN-γ, and TNF-α were significantly increased in patients with COVID-19 compared to controls. In contrast, IFN-α was reduced in the COVID-19 group; these data suggest a possible relationship between vitamin D concentration, immune system function, and risk of infection with COVID-19 [122].

During the recent COVID-19 outbreak, individuals suffering from vitamin D and selenium deficiency developed acute respiratory tract infections. Vitamin D enhances immunity by stimulating the production of antimicrobial peptides. Furthermore, vitamin D also prevents cytokine storms by reducing the secretion of inflammatory cytokines by immune cells. Selenium, on the other hand, boosts the cytotoxic effects of immune cells [111]. Interestingly, vitamin D receptors and other related enzymes are expressed by both innate and adaptive systems, probably explaining the immunomodulatory properties of vitamin D. Therefore, vitamin D deficiency is often linked with allergies, infections, and autoimmune disorders [123].

A randomized, active-control, double-blind study in HIV patients showed that high-dose vitamin D supplementation (120,000 IU/month) reduced T-cell activation/exhaustion and monocyte activation, preventing abnormal immune activation and immune response in HIV patients, a leading cause of comorbidities in HIV patients [104]. In a clinical trial, sarcopenia patients aged ≥65 were given 20 g of whey protein, 3 g of leucine, and 800 IU of vitamin D twice daily for 13 weeks. After the intervention period, IL-6 and IL-1Ra levels increased, but IL-8 levels were reduced. The study concluded that vitamin D and whey protein supplementation may reduce the immune response in sarcopenia patients [124].

Interestingly, vitamin D prevents autoimmunity by preventing the activation of T-regulatory cells. A double-blind, placebo-controlled study in 60 subjects showed that high-dose vitamin D for 12 weeks (140,000 IU/month) significantly increased the numbers of T_reg_ cells without changing the numbers of other immune cells such as monocytes, T and B cells, NK cells, and dendritic cells. The high dose of vitamin D was found to be safe and did not cause any side effects [125].

### 4.3. Folic Acid

Folic acid is also known as vitamin B9. The important natural sources of this vitamin are peanuts (246 μg/100 g), sunflower seeds (238 μg/100 g), asparagus (149 μg/100 g), lettuce (136 μg/100 g), etc. The nutritional recommendations of the National Institutes of Health (USA) describe that the tolerable upper intake of folic acid levels for adults are regarded as 1000 μg/day [126].

A cellular experiment was designed to evaluate the role of folic acid in the proinflammatory and antiviral molecular pathways of B-lymphocytes infected with a modified live vaccine. It was established that folic acid could modulate the response of B-lymphocytes to improve the molecular pathways of their antiviral and innate immune proinflammatory responses [127]. A study that identified key genes and effective compounds through the analysis of effective compounds and drug targets against COVID-19 showed that folic acid acts on SARS-CoV-2 N through molecular docking and antagonizes the inhibitory effect of the protein on the RNA interference pathway, which is the perspective of the therapeutic use of FC supplements [128]. Importantly, these results were confirmed by the more severe clinical course of COVID-19 and the worse outcomes associated with micronutrient deficiencies, especially low levels of folic acid [129]. However, isolated clinical folic acid deficiency cases are extremely rare; most of the symptoms coincide with cobalamin deficiency; the proliferative effects of folic acid may increase the risk of cancer [130]; it is suggested to follow practical advice on micronutrient provision and monitoring during nutritional support [131]. To conclude, knowledge of the main mechanisms of the interaction between the virus and the host has expanded the use of folic acid supplementation and the opportunities for developing new medications.

### 4.4. Magnesium

Magnesium is an essential micronutrient required for various physiological processes, and there is increasing evidence that it is needed to support the normal functioning of the body’s immune system. The main natural dietary sources of magnesium are bananas, nuts, black beans, whole grains, flaxseed, green vegetables, and pumpkin seeds [132]. Hypomagnesemia can lead to various chronic inflammatory diseases [76].

Several studies have shown that magnesium may have a protective role against COVID-19 by reducing lung inflammation [133]. According to the results of this study, magnesium levels were lower in patients with more severe symptoms of COVID-19, and the median magnesium level in patients with severe symptoms of COVID-19 was 38.33 mg/L. In contrast, the median magnesium level in the group with mild symptoms was 39.46 mg/L, giving a *p* value of 0.002 between the two groups. This study shows that higher magnesium levels may protect against severe symptoms of COVID-19 [134]. Mechanisms include its calcium channel-blocking effects, which lead to inhibition of interleukin-6, CRP, nuclear factor Kβ, and other potentially disruptive factors. In addition, magnesium deficiency is associated with increased IL-6, a proinflammatory cytokine and a likely target for the treatment of COVID-19 [135].

Magnesium contributes to the regulation of the immune response by affecting the cells of the innate and adaptive immune systems. The low magnesium content increases the reactivity to various immune reactions and participates in the pathophysiology of many common chronic diseases; it increases the level of cytokines and the oxidation of granulocytes, stimulates phagocytes, activates endothelial cells, and thus, promotes inflammation [136].

Importantly, altered magnesium homeostasis may contribute to long-term COVID syndrome and exacerbate it after the acute phase; increasing reports describe persistent and long-lasting effects after acute COVID-19 affecting the lungs, heart, brain, and gastrointestinal system, as well as neurological manifestations of magnesium deficiency such as insomnia, anxiety, depression, dizziness, and headache. Hence, assessing and correcting magnesium levels is essential for facilitating recovery [137].

There are many formulations of magnesium, including inorganic (oxide, sulfate, and chloride) and organic (citrate, malate, and taurate) compounds. The doses of administered Mg2+ varied widely among studies (from 100 to 1000 mg/day) [138]. As it was observed by Fiorentini et al. [138], toxic hypermagnesemia occurred if oral magnesium doses were higher than 2500 mg/day. Generally, magnesium was regarded as a safe and cost-effective mineral that could help restore the damaged homeostatic equilibrium of the human body [137].

### 4.5. Zinc

The irreplaceable trace element zinc is of crucial importance for many physiological processes; it plays a significant role in maintaining immune homeostasis by affecting the functional capacity of the cells of the innate and adaptive immune systems and has a regulatory effect on the production of cytokines and antibodies and the activity of the complement system, so the deficiency of this element can contribute to the violation of immune function and thus have a significant impact on health [139]. The richest source of zinc is found in foods obtained from animals, such as seafood, meat, and milk products [140]. Some cereal-based products and vegetables were also regarded as appropriate natural sources of zinc. A meta-analysis and systematic review conducted by Jafari et al. aimed to investigate the effect of zinc supplementation on immune factors in a total of 35 randomized controlled trials with 1995 participants. It was observed a significant decrease in circulating serum CRP (weighted mean difference (WMD): −32.4; 95% confidence interval (CI): from −44.45 to −19.62, *p* < 0.001), high sensitivity C reactive protein concentrations (WMD: −0.95; 95% CI: from −1.01 to −0.89, *p* < 0.001), neutrophil levels (standard mean difference (SMD): −0.46; 95% CI: −0.90 to −0.01, *p* = 0.043), and CD4 levels (WMD: 1.79; 95% CI: 0.57 to 3, *p* = 0.004) after zinc supplementation [141].

A previous study showed that the expression of angiotensin-converting enzyme 2 (ACE2) regulates the signaling of reactive oxygen species and NF-κB in a human lung cell line; zinc supplementation enhances the inhibitory effect and not only destroys the balance of the immune response but also affects the expression of ACE2 receptors, which are necessary for penetration into target cells for ACE2-associated diseases such as COVID-19 and should be considered for treatment. Moreover, zinc has the potential to restore depleted immune cell function or improve normal immune cell function and may also act synergistically when used concurrently with standard antiviral therapy [142].

A meta-analysis of trace element zinc levels in COVID-19 patients and controls, according to severity and nonseverity, as well as nonsurvivors and survivors, using a random-effects model from 11 studies, showed that the overall SMD in zinc levels between patients with COVID-19 and controls was −0.83 (−1.19 to 0.46, *p* < 0.05), indicating that patients with COVID-19 had significantly lower zinc levels. In addition, the difference in zinc levels between severe and nonsevere patients, or survivors and nonsurvivors, was assessed, and severe COVID-19 patients had significantly lower zinc levels than nonsevere COVID-19 patients (SMD: −0.47, 95% CI: −0.75 to −0.18, *p* < 0.05). However, zinc levels in nonsurvivors were not significantly different from those in survivors with COVID-19 (SMD: −1.46, 95% CI: −3.98 to 1.06, *p* < 0.05) [143].

A randomized clinical trial showed that zinc supplementation during severe pneumonia did not improve recovery from respiratory disorders and only showed marginal improvement in the severity of symptoms. The study outcomes do not support using zinc as an adjunct therapy for treating severe pneumonia in zinc-replete children [144]. A randomized controlled trial in children (*n* = 512) aged 6–23 months demonstrated that zinc supplementation (7 mg/day) combined with a multiple micronutrient powder for nine months did not affect cytokine secretion or the number of T cells. However, zinc supplementation altered the numbers of lymphocytes and eosinophils [145]. Daily ingestion of zinc supplements (40 mg/day in adults) was generally considered safe [146].

It was considered zinc as a preventive and adjunctive therapy against COVID-19 for groups at risk of zinc deficiency. In addition, existing zinc deficiency exacerbates inflammatory disease, whereas prophylactic zinc supplementation reduces the severity of the disease or even prevents its development. Research evidence highlights the important role of zinc in immune function, but there are also conflicting results showing that high doses of zinc cause immune dysfunction and related health problems. Regular assessment of zinc status should be performed, and such knowledge should help link existing zinc deficiency to the development and severity of certain diseases [147].

### 4.6. Selenium

Selenium is a trace element that is found in the body in minimal concentrations but can play an important role in the functioning of the immune system, protecting the body from infections, especially those of viral origin. It takes part in the enzymatic functions of catalyzing glutathione peroxidase, deiodinase, and thioredoxin reductase, serves as an antioxidant, and protects cell membranes and organelles from peroxide destruction [148]. Selenium may be a potential agent for preventing viral infections, including coronavirus, according to the mechanism of enhancing the proliferation of NK cells. T-lymphocytes also have a positive effect in combination with vitamins D and E [149]. Considering that increased oxidative stress and excessive production of inflammatory cytokines are critical components of coronavirus disease, selenium reduces the level of viral infection, reduces oxidative stress and inflammation, strengthens the immune system, and is necessary for critically ill patients. Moreover, selenium deficiency is often associated with the severity and mortality of the disease, providing new insight into the relationship between selenium deficiency and the severity of COVID-19 [150].

The study has shown that selenium deficiency reduced the expression of some antioxidant selenoproteins, thereby additionally inducing the accumulation of reactive oxygen species and oxidative stress, reducing the activity of glutathione peroxidase (*p* < 0.05), thioredoxin reductase, and catalase, as well as the inhibitory capacity for hydroxyl radicals, and increasing (*p* < 0.05) the content of malondialdehyde and nitrogen oxide. Its deficiency increased (*p* < 0.05) NF-κB transcription factor expression and regulated inflammatory cytokines. Se deficiency increased (*p* < 0.05) the expression of IL-6, IL-8, IL-12, IL-17, and cyclooxygenase-2 and decreased (*p* < 0.05) the expression of IL-10, IL-13, and TGF-β, which indicates the regulation of selenoproteins of oxidative stress and inflammation [151].

Research by Fath et al. focused on selenium supplementation, which reduces the ability of SARS-CoV-2 to infect human cells by increasing T cells, especially CD4+ T cells, the percentage of NK cells, and subsequently NK cell cytotoxicity [152]. The main sources of organic selenium are beef/sheep liver, red meats, poultry, eggs, seafood, dairy products, some fungi, brazil nuts, broccoli, and whole grain foods [111]. WHO recommends that the daily intake of selenium by adults should be 40–70 µg/day, depending on body condition and gender (weight, pregnancy in women, etc.) [111]. It should be noted that selenium in doses above 400 µg/day demonstrates harmful actions [149]. Recently, Vahidi et al. [153], during a study of the anticancer properties of selenium, concluded that its nano-formulations could be developed for targeted delivery to tumors and that they would significantly decrease the dosage of selenium to minimize the possible adverse effects.

Considering the research results, selenium supplementation seems promising, so blood levels should be carefully monitored. While taking selenium supplements, it is important to follow the appropriate dosage to minimize potential toxic side effects.

It could be concluded that certain vitamins and minerals play a pivotal role in boosting immune function. A very important aspect of their applications in the last 3 years is that micronutrients are important immunomodulators for COVID-19 management.

## 5. Probiotics, Prebiotics, Synbiotics, and Immunity

Probiotics are described as live bacteria that, when consumed adequately, confer a health benefit on the host [154]. The first records of ingestion of live bacteria by humans were over 2000 years old. Prebiotics have been defined as nondigestible food ingredients that beneficially affect the host by selectively stimulating the growth and/or activity of one or a limited number of bacterial species already resident in the colon and thus attempt to improve host health [155]. Various molecules can be prebiotics, but most are dietary fibers. They serve as substrates for probiotic commensal bacteria that release short-chain fatty acids in the intestinal tract along with some other metabolites. A synbiotic contains both prebiotics and probiotics, which can stimulate and increase the survival of autochthonous microbiota and probiotic bacteria, particularly lactobacilli and bifidobacteria.

The best-studied and best-characterized probiotics belong to the lactic acid bacteria group, which mainly includes the genera *Lactobacillus*, *Bifidobacterium*, *Leuconostoc*, *Lactococcus*, *Pediococcus*, and *Streptococcus* [156]. *Lactobacillus* strains can enhance the humoral immune response to infections [157] and vaccination [158].

Probiotics impact intestinal microbiota balance, making it benign and promoting the absorption of active substances, improving human physiological functions. Thus, severe dysbiosis due to reducing the concentration of *Lactobacillus* spp., *Bacteroides* spp., etc., in patients with rheumatoid arthritis led to an overgrowth of pathogenic microorganisms [159]. Probiotics promote the digestion and absorption of nutrients and maintain gut microbiota homeostasis. As beneficial natural inhabitants of the human microbiome, probiotics play an important role in producing various nutrients, preventing infectious diseases caused by intestinal pathogens, and modulating the immune response [160]. Probiotics are chosen as alternatives because they act as natural immune enhancers [161]. The mechanisms by which probiotic strains modulate the immune response are not completely known. Probiotics affect innate immunity by preventing bacterial adherence and translocation and improving the mechanisms of pathogen destruction. Prevention of the initial step of tissue colonization by pathogens may improve the treatment of some infectious diseases. For example, *Saccharomyces boulardii* significantly reduces some adverse effects of *Helicobacter pylori* eradication therapy by preventing adherence of this pathogen to the mucous membrane of the stomach [162].

Probiotic strains can produce bacteriocins, contributing to the antimicrobial activity of the gut microbiome [163]. Species from the genus *Lactobacillus* release various bacteriocins that inhibit taxonomically related gram-positive bacteria. Some are active against a much wider range of gram-positive and gram-negative bacteria, yeasts, and molds. Adhesion and invasion of an intestinal epithelial cell line (Intestine 407) by adherent invasive *E. coli* isolated from patients with Crohn’s disease (CD) were substantially diminished by co- or pre-incubation with the probiotic strain *E. coli* Nissle 1917 [164]. *E. coli* Nissle is known to secrete microcins to antagonize its competitors [165]. These findings have shown that probiotics avert epithelial injury induced by pathogenic bacteria, contribute to an improved mucosal barrier, and limit enteric pathogen access.

Probiotic bacteria can stimulate enterocytes to produce cytoprotective substances, such as heat shock proteins and antimicrobial peptides, e.g., defensin and mucin. The induction of defensins by probiotics might be an interesting new therapeutic strategy to strengthen innate defense mechanisms. *E. coli* Nissle 1917, *L. fermentum*, *L. acidophilus* PZ 1138, *Pediococcus pentosaceus,* and other probiotics activate the human-beta defensin 2 promoter, inducing the intestinal barrier defense system without provoking inflammation in patients, while the induction of human-beta defensin 2 expression by the pathogenic strains *Salmonella* ssp. and *H. pylori* provokes inflammation [166]. It was investigated and found that flagellin is the major stimulatory factor of *E. coli* Nissle 1917 [164].

Probiotics can downregulate the expression of proinflammatory cytokines and ameliorate intestinal inflammatory diseases. They can suppress the nuclear NF-κB signaling pathway [167], possibly related to alterations in mitogen-activated protein kinases and pattern recognition receptor pathways. Probiotics can also inhibit the binding of LPS to the CD14 receptor, reducing the overall activation of NF-κβ and produce proinflammatory cytokines [168]. Kim et al. showed that conjugated linoleic acids produced by probiotics interact with IkB kinase and heat shock protein 90 to activate the NF-kB signaling pathway in human gastric epithelial cells infected with *H. pylori* [169]. Lv et al. have shown that probiotics generally cannot eliminate *H. pylori*, although they decrease the density of colonization, thereby maintaining lower levels of *H. pylori* in the stomach [170].

Regarding the adaptive immune response, numerous studies have shown that probiotic impacts seem to be variable. Many probiotic strains seem capable of stimulating IgA production by B cells. In children with viral gastroenteritis caused by rotavirus, probiotics such as *L. rhamnosus* have been shown to stimulate rotavirus-specific IgA antibody responses. A stronger significant inverse correlation was observed between small intestinal HRV IgA titres and mean fecal scores in Vac + Pro piglets compared with vaccinated probiotic-colonized piglets [171]. Thus, reinfections can be partly prevented by probiotic intestinal cell-mediated responses. *Pediococcus acidilactici* K15, as well as LAB, was found to be the most effective in inducing IgAs, which are mainly induced via IL-10, as well as IL-6, secreted by K15-stimulated dendritic cells. In a clinical trial, an increase in salivary sIgA concentration by K15 ingestion was confirmed [172]. The health benefits ascribed to one strain of probiotic may not be obligatory when applied to another strain [173]. Díaz-Ropero et al. showed that the consumption of *L. fermentum* CECT5716 enhanced the production of Th1 cytokines by spleen cells and increased the IgA concentration in feces. Therefore, this probiotic has an immunostimulatory effect. However, the consumption of *L. salivarius* CECT 5713 induced IL-10 production by spleen cells, demonstrating the anti-inflammatory effect [174].

The potential of probiotics to boost health benefits has been reported, as they can regulate allergic reactions, alleviate inflammatory bowel disease, reduce tumor growth in some cancer models, prevent colon cancer, control the levels of blood cholesterol, and protect hosts from bacterial and viral infections [175]. The prominent probiotic *Escherichia coli* NISSLE 1917 is used in the treatment of intestinal diseases such as diarrhea and inflammatory bowel disease because it has high adhesive properties and demonstrates immunomodulation and anti-inflammation abilities [176]. The effects of *Bifidobacterium infantis* supplementation in an inflammatory bowel disease mouse model demonstrated relief of intestinal epithelial injury and increased production of cytokines, such as transforming TGF-β1, IL-10, and IL-35, which play an immunosuppressive role in maintaining the body’s immune tolerance [177]. The research of Xiao Joe et al. using principal component analysis and cluster analysis heatmaps showed that the novel candidate probiotic mixture is effective against the nervous necrosis virus as a consequence of stimulation of innate and adaptive immunity [178]. Probiotics from the *Bifidobacterium* and *Lactobacillus* genera have exhibited feasible antiviral activities against rotavirus [179] and enterovirus [180]. Randomized controlled trials found that supplementation with probiotics or prebiotics as adjuvant therapy for COVID-19 might positively affect the outcome of COVID-19, and improvements in fatigue, anosmia, breathlessness, nausea, vomiting, and other gastrointestinal symptoms were reported [181,182]. The administration of probiotics in the diet of patients with gastrointestinal symptoms related to COVID-19 and those with mild-to-moderate systemic symptoms can be an alternative for preventing cytokine storms [182].

Prebiotics can induce a microbiota-independent effect by directly acting on gut-associated epithelial and innate immune cells through Toll-like receptors. Studies in the animal model showed that the prebiotics inulin/oligofructose primarily activated immune cells in Peyer’s patches, including IL-10 production and NK cell cytotoxicity. They modulated the concentration of secretory IgA in the ileum and caecum [183]. Intestinal DCs are located within gut-associated lymphoid tissue and express pattern recognition receptors such as TLR-2 and TLR-4. Prebiotics or their fermentation byproducts can bind to these receptors and trigger the maturation process involving upregulation or downregulation of membrane CD83, CD86, HLA-DR, and DC-SIGN molecules and induce the secretion of cytokines. The activation of DCs through particular pattern recognition and immunity receptors defines the polarization of effector T cells to either the T-helper 1 (Th1), Th2, Th17, or Treg phenotype [184]. Some authors showed that butyrate, acetate, and soybean oligosaccharides could enhance the activity of NK cells in comparison with the control group [185,186]. The symbiotic combination of enriched inulin with probiotics such as *Lactobacillus rhamnosus* GG and *Bifidobacterium lactis* Bb12 enhanced NK cell activity in the blood [185]. Studies in poultry flocks indicated that the probiotic strain *Enterococcus faecium* combined with prebiotics inulin and fructooligosaccharides might inhibit pathogen proliferation, insomuch as significantly extending the lag time for pathogenic *E. coli* O1/O18 and *Salmonella enterica* serotype *Enteritidis* strains [187].

The global nutraceutical market, including the probiotics market, is one of the fastest-growing segments of the food industry. Widespread use of probiotic products due to growing reports about the health demands to estimate their bioavailability, safety, and side effects. The European Food Safety Authority has developed guidelines for the safety assessment of probiotics as a food additive (FEEDAP, 2014). Currently, probiotic products have been commercially regulated in three forms: functional foods (fermented foods), dietary supplements, and drugs (pharmaceuticals) [188]. Probiotics are a priori nonpathogenic; therefore, they are never supposed to cause or potentiate any disease in humans. The safety of medicinal probiotics is still being discussed. The safety of probiotics was confirmed as apparent due to the absence of toxicity by randomized trials in different target populations, conditions, and age groups in a specific clinical scenario [189,190,191,192,193].

Probiotics are categorized in accordance with the site of action (the oral, upper respiratory, or gastrointestinal tract). As it is known, the dosage form must be in adequate amounts to confer a health benefit. The dose of a drug is indirectly proportional to its bioavailability. The viability of probiotic bacteria is a requirement to meet the definition of a probiotic. It is questionable when probiotics are exposed to harsh environments during processing (i.e., dehydration), storage, and delivery to their site of action. Their efficacy is dependent upon delivering an adequate dose throughout the product’s shelf life. The World Health Organization (WHO) and the Food and Agriculture Organization (FAO) reported that the viability of probiotics in food products should be counted at least at 106 colony-forming units /g alive microbial cells [194,195,196,197]. According to the majority of human clinical studies, probiotic dosages typically range between 107 and 1011 colony-forming units /day. The appropriate dose, duration, and composition of a synbiotic needed to confer a health benefit are likely to be specific to outcome and baseline host target site microbiota, as well as coexisting environmental factors such as medication, habitual diet, and host genetic factors [198].

Despite the tremendously beneficial effects of probiotics and prebiotics on health, the WHO and FAO reported [199] that they might have some side effects, and safety issues may arise due to the use of living microbial cells [200]. The topic of probiotic safety is still being discussed [201,202]. Evidence from clinical trials is mixed and controversial [194]. The long-term colonizing probiotics have potential risks, such as that the probiotic could displace a microbe performing an important function; negatively impact the structure and function of the surrounding microbiota; and if the normal gut barrier is breached, the probiotic could access the systemic circulation, resulting in invasive infection [203]. Suez J. and colleagues [204] found that resident gut bacteria in a subset of individuals resist the mucosal presence of probiotic strains, limiting their modulatory effect on the microbiome. Evidence of probiotic gut mucosal colonization efficacy remains sparse. The elicited cytokine production via probiotic supplementation might cause inordinate immunological effects. Theoretically, probiotic administration might lead to auto-immune disease development or certain inflammations [205]. Building a strong safety profile for probiotic strains will solidify their use in individuals that can benefit the most from microbial modulation.

As could be seen from the results of the experimental and clinical studies described above, a lot of probiotics, prebiotics, and synbiotics are very useful for improving immune function. When probiotics are mainly alive, useful bacteria, prebiotics are regarded as dietary polysaccharides or oligosaccharides of plants. A symbiotic relationship contains both of these groups and can increase the survival of autochthonous microbiota and probiotic bacteria (mainly lactobacilli and bifidobacteria).

## 6. Conclusions

The body’s immune system fights against a huge number of pathogens and antigens and plays a central role in our survival. Therefore, enhancing the immune system’s ability is highly beneficial in preventing many health disorders. Some medicinal plants and different natural ingredients have been effectively used to augment immunity and prevent infections since ancient times. In today’s era, there have been a lot of experimental and clinical studies demonstrating that some natural ingredients from plants, fungi, animals, and microorganisms can directly modulate multiple signaling pathways, alter gene expression, and change the secretion of pro- and anti-inflammatory cytokines. Moreover, these bioactive natural ingredients increase the activity of numerous immune cells, including neutrophils, basophils, mast cells, macrophages, NK cells, and lymphocytes, thereby increasing immune functions.

The present review is an attempt to explore the health-promoting effects of certain natural ingredients from medicinal plants and different dietary sources. Some of these natural ingredients have been used by humankind since time immemorial. Nowadays, a lot of preclinical and clinical studies have also found that many of these traditional sources as well as some comparatively recently discovered health-promoting agents (vitamins, minerals, polyphenols, terpenoids, polysaccharides, melatonin, pre- and probiotics) are efficacious in boosting immunity and safe to consume. However, there is a lack of clinical trials for some of the natural ingredients covered in the present review. Hence, future research must be directed toward finding their efficacy, an appropriate dose range, and dosage forms, as well as mechanisms of action, for further promoting their use as immunity-enhancing agents.

## Data Availability

Data sharing not applicable.
